# The effect psychobiotics on anxiety symptoms

**DOI:** 10.1192/j.eurpsy.2023.975

**Published:** 2023-07-19

**Authors:** Y. Denysov, G. Putyatin, S. Moroz, V. Semenikhina

**Affiliations:** 1 Psychiatry, psychotherapy, addictology and medical psychology, Donetsk national medical university, Kropyvnitskyi; 2 Psychiatry, Municipal Enterprise “Dnipro Multiprofile Clinical Hospital for the Provision of Psychiatric Care of Dnipropetrovsk Regional Council, Dnipro, Ukraine

## Abstract

**Introduction:**

Psychobiotics are a group of probiotics that affect the central nervous system related functions and behaviors mediated by the gut-brain-axis via immune, humoral, neural, and metabolic pathways to improve not only the gastrointestinal function but also the antidepressant and anxiolytic capacity.

**Objectives:**

An assessment of psychobiotic and anti-anxiety effects a probiotic supplement containing Lactobacillus Plantarum CECT7485 and Lactobacillus Brevis CECT7480 (PLANTARUM) in patients with anxiety undergoing treatment with selective serotonin reuptake inhibitors (SSRI) antidepressants.

**Methods:**

Sixty patients with mixed anxiety and depressive disorder (according to ICD-10 diagnostic criteria F41.2) were included in an 8-week open label study. Thirty participants received either SSRI antidepressants with PLANTARUM at a dose of 1.0 × 10^9^ CFU once per day and thirty patients received SSRI antidepressants only. The severity of anxiety symptoms was assessed using Hamilton Anxiety Rating Scale (HAM-A) and General Anxiety Disorder Scale (GAD-7).

**Results:**

After 8 weeks intervention, a significant reduction of HAM-A total score (from 37,8±5,3 to 23,6±4,4) was detected in patients with anxiety who prescribed SSRI antidepressants and PLANTARUM (p˂0,05), compared with participants who didn’t receive probiotics (p>0,05). Also, a significant reduction of GAD-7 total score (from 21,7±3,3 to 12,5±2,4) was detected in patients with anxiety symptoms who received SSRI antidepressants and PLANTARUM (p˂0,01), compared with patients who didn’t intake probiotics (p>0,05).

**Conclusions:**

The present data illustrated that probiotic supplement PLANTARUM is a feasible for adjunctive to SSRI antidepressants intervention for anxiety treatment.

**Disclosure of Interest:**

None Declared

